# Predictive value of neutrophil to lymphocyte ratio on acute kidney injury after on-pump coronary artery bypass: a retrospective, single-center study

**DOI:** 10.1007/s11748-022-01772-z

**Published:** 2022-02-01

**Authors:** Zhang Guangqing, Cheng Liwei, Ling Fei, Zheng Jianshe, Zeng Guang, Zhu Yan, Cao Jianjun, Tian Ming, Chen Hao, Liu Wei

**Affiliations:** 1 Department of Cardiothoracic Macrovascular Surgery, Sinopharm Dongfeng General Hospital, Hubei University of Medicine, Shiyan, 442008 Hubei People’s Republic of China; 2 Department of Pulmonary and Critical Care Medicine, Hanchuan People’s Hospital, Xiaogan, 432300 Hubei People’s Republic of China; 3 School of Nursing, Hubei University of Medicine, Shiyan, 442000 Hubei People’s Republic of China; 4 Department of Nephrology, Wuhan Fourth Hospital, Puai Hospital, Tongji Medical College, Huazhong University of Science and Technology, 430030, Wuhan, Hubei People’s Republic of China

**Keywords:** Acute kidney injury, Neutrophil to lymphocyte ratio, Coronary artery bypass grafting, Cardiopulmonary bypass

## Abstract

**Objective:**

To investigate the predictive value of preoperative neutrophil to lymphocyte ratio (NLR) on acute kidney injury (AKI) after on-pump coronary artery bypass (ONCAB).

**Methods:**

Patients who underwent elective ONCAB for coronary heart disease were included. NLR was calculated according to the results of preoperative routine blood test, patients were divided into non-AKI and AKI groups, and the differences in clinical baseline data between the two groups were compared.

**Results:**

A total of 154 patients were included in this study, including 57 (37%) with postoperative AKI and 97 (63%) without AKI. Compared with the patients in non-AKI group, those in AKI group had higher NLR (2.63 (1.83, 3.505) vs. 2.06 (1.7, 2.56), *p* = 0.002), higher serum creatinine (78 (67, 98.5) vs. 70.9 ± 16.8 umol/L, *p* < 0.001), longer cardiopulmonary bypass time, and longer aortic cross clamp time. After dividing patient into tertiles based on NLR, those with higher NLR had higher risk of postoperative AKI than those with lower NLR (30% vs. 25% vs. 55.8%, *p* for trend = 0.003). Patients in Tertile2 and Tertile3 had higher NLR compared to those in Tertile1 (*p* < 0.05); multivariate logistic regression analysis showed patients with elevated preoperative NLR and blood creatinine had higher risk of postoperative AKI. ROC curve showed that patients’ preoperative NLR combined with blood creatinine had better predictive value for postoperative AKI.

**Conclusion:**

Elevated preoperative NLR is associated with AKI after ONCAB, and had prognostic utility independent of other recognized risk factors.

**Supplementary Information:**

The online version contains supplementary material available at 10.1007/s11748-022-01772-z.

## Introduction

Globally, coronary heart disease is the most common cause of death [1]. Coronary artery bypass grafting (CABG) remains the mainstay of treatment for patients with severe multivessel disease, especially for those combined with left main stem lesions in coronary artery disease, in which CABG can reconstruct blood flow and relieve myocardial ischemic and hypoxic symptoms. Despite the great improvements in surgical approaches and supervised treatment in cardiac surgery, the incidence and mortality associated with acute kidney injury (AKI) has not been significantly reduced in recent years [2]. Several studies have shown that AKI is a common complication after CABG, up to 5–30%, of which 1–2% of patients may require renal replacement therapy [3]. AKI contributes to patients’ increased complication rates and morbidity and mortality, prolonged hospital stays and increased medical costs [4]. Even a slight increase in postoperative blood creatinine may increase morbidity and mortality [5]. AKI after cardiac surgery is closely related to inflammation, ischemia and reperfusion injury, neurohormonal activation, and oxidative stress [6]. Therefore, early warning and identification of patients at high risk of AKI after CABG is extremely important to guide individualized treatment and improve patient prognosis. Lymphocyte apoptosis is a prevalent phenomenon in the inflammatory response, and neutrophils as phagocytic cells are involved in the complex mechanisms regulating inflammatory and immune responses in the organism. In inflammatory states, neutrophil to lymphocyte ratio (NLR) can better reflect to the various intercellular imbalance in the body. Studies showed that NLR had the positive correlation and synchronous changes with various classical inflammatory indices, indicating the state of renal function during the inflammation and disease processes [7]. However, existing studies have shown the conflicting predictive value of preoperative NLR for postoperative AKI in patients undergoing CABG alone [6, 8]. In this study, we aimed to investigate the correlation between preoperative NLR and postoperative AKI after ONCAB, and the combined predictive value of preoperative NLR with other indicators.

## Materials and methods

### Study population

Patients undergoing ONCAB for coronary artery disease at Sinopharm Dongfeng General Hospital from October 2016 to June 2021 were retrospectively analyzed. All patients underwent CABG under mild hypothermia cardiopulmonary bypass. Myocardial protection was performed in all cases by intermittent antegrade perfusion of cold blood cardioplegic solution. Extracorporeal rewarming was started after completion of proximal anastomosis and stopped when nasopharyngeal temperature reached 36–37 °C, rectal temperature reached 35–36 °C, hemoglobin ≥ 80 g/L, and hematocrit ≥ 24%. A single aortic cross clamp (ACC) technique was used in all cases.

*Inclusion criteria* (1) age ≥ 18 years; and (2) diagnosis confirmed by cardiac ultrasound and coronary angiography, with clear indications for CABG.

*Exclusion criteria* (1) incomplete clinical data; (2) preoperative renal replacement therapy or maintenance hemodialysis; (3) simultaneous cardiac combined renal surgery; (4) preoperative infectious status or history of AKI within the past 3 months; (5) chronic autoimmune disease with hormone therapy; (6) severe heart failure; (7) renal transplantation; and (8) postoperative death.

### Methods

Baseline clinical data were collected from all eligible patients, including gender, age, underlying disease, comorbidities, surgical approach, intraoperative recording of cardiopulmonary bypass time, ACC time, changes in blood creatinine and urine volume during the 7 days after surgery, and preoperative laboratory tests [blood routine test and NLR, blood type, coagulation function, liver and kidney function, blood lipids, brain natriuretic peptide (BNP), troponin, and left cardiac ejection fraction (LVEF) assessed by echocardiography].

Relevant definitions: (1) the diagnosis of chronic kidney disease was referred to the Kidney Disease Outcomes Quality Initiative (KDOQI) guidelines [9]. (2) AKI was diagnosed according to the modified KDIGO guidelines [10] by meeting any of the following: (i) rapid decline in renal function within 48 h after surgery with an absolute increase in serum creatinine ≥ 26.5 umol/L (0.3 mg/dl); (ii) increase in serum creatinine to ≥ 1.5 times the basal value at 7 days; (iii) urine volume < 0.5 mL/(kg h) with duration > 6 h.

### Statistics

Continuous normal distribution variables were expressed as mean ± standard deviation in each subgroup. The two samples were compared by independent sample *t* test; continuous variables of non-normal distribution were expressed as median concentration and quartile distance. Mann–Whitney *U* test is used for the comparison between the two groups, and Kruskal–Wallis h test is used for the comparison between multiple groups. Categorical variables were expressed as percentage, and the chi-square test was used for the comparison between groups. The analysis of linear trends was used to assess the correlation between increasing the NLR levels and risk of AKI after the sample was divided into tertiles based on the distribution of controls. Multivariate logistic regression analysis was used to further analyze the correlation between NLR and AKI. The best cut-off value of NLR for predicting AKI was analyzed by receiver operating characteristic (ROC) curve. Differences with a *p* value of < 0.05 (two-tailed) were considered to be statistically significant. Analysis was performed using R version 3.6.0. GraphPad prism 8 and Photoshop version 6.0 software are used for graphic production.

## Results

### Characteristics of enrolled patients

According to the inclusion and exclusion criteria, 154 patients were finally included in this study. Among them, 66.9% were male and 33.1% were female; 34.4% cases were under 60 years old and 65.6% were over 60 years old. 37% patients had previous diabetes mellitus, 74% had hypertension, 7.1% had atrial fibrillation, 20.1% had cerebrovascular disease, 5.8% had chronic kidney disease, 46.1% had myocardial infarction, and 30.5% had heart failure.

### Comparison of baseline data between AKI and non-AKI patients

We divided patients into AKI group (*n* = 57, 37%) and non-AKI group (*n* = 97, 63%) to determine whether there were differences in clinical baseline data between the two groups. Compared with the patients in non-AKI group, those in AKI group had higher proportion of chronic kidney disease (2.1% vs. 12.3%, *p* = 0.009), significantly lower lymphocyte count (1.5 (1.1, 1.9) vs. 1.7 (1.5, 2.1) × 10^9^/L, *p* = 0.005), higher NLR (2.63 (1.83, 3.505) vs. 2.06 (1.7, 2.56), *p* = 0.002), higher serum cystatin C (1.195 (0.965, 1.385) vs. 1.01 (0.875, 1.175) mg/L, *p* = 0.003), higher brain natriuretic peptide (240.9 (108.3, 511.4) vs. 118.2 (47.725, 482.175) pg/ml, *p* = 0.041), higher serum creatinine (78 (67, 98.5) vs. 70.9 ± 16.8 umol/L, *p* < 0.001), higher proportion of patients with decreased LVEF (5.7% vs. 29.2%, *p* < 0.001), longer cardiopulmonary bypass time (125 (108, 130) vs. 114.5 (93, 125) min, *p* = 0.004), and longer ACC time (68 (58.5, 81) vs. 63 (53, 70.5) min, *p* = 0.019). However, there was no significant difference between the two groups in terms of age, gender, or comorbidities, including diabetes, hypertension, atrial fibrillation, myocardial infarction, cerebrovascular disease, and heart failure (*p* > 0.05), while no difference was found between the two groups in terms of white blood cell count, neutrophil count, albumin, blood lipids, uric acid, and ultrasensitive troponin (*p* > 0.05), as shown in Table 1.

**Table 1:** Demographic and clinical characteristics of the patients at baseline

Group	No-AKI	AKI	*P*-value
	97	57	
Age-no., %			
≦60 years	33(34.0%)	20(35.1%)	0.893
> 60 years	64 (66.0%)	37(64.9%)	
Gender-no., %			0.169
Male	61 (62.9%)	42 (73.7%)	
Female	36 (37.1%)	15 (26.2%)	
Diabetes-no., %			0.704
No	60 (61.9%)	37 (64.9%)	
Yes	37 (38.1%)	20 (35.1%)	
Hypertension-no., %			0.492
No	27 (27.8%)	13 (22.8%)	
Yes	70 (72.2%)	44 (77.2%)	
Previous AF-no., %			0.487
No	89 (91.8%)	54 (94.7%)	
Yes	8 (8.2%)	3 (5.3%)	
Previous stroke-no., %			0.827
No	78 (80.4%)	45 (78.9%)	
Yes	19 (19.6%)	12 (21.1%)	
Previous MI-no., %			0.926
No	52 (53.6%)	31 (54.4%)	
Yes	45 (46.4%)	26 (45.6%)	
CKD-no., %			0.009
No	95 (97.9%)	50(87.7%)	
Yes	2 (2.1%)	7 (12.3%)	
Previous HF-no., %			0.345
No	70 (72.2%)	37 (64.9%)	
Yes	27 (27.8%)	20 (35.1%)	
WBC count, × 10^9^/L	5.9 ± 1.4	5.9 (5.3, 6.85)	0.477
Neutrophil count, × 10^9^/L	3.6 ± 1.0	3.9 (3.1, 4.6)	0.101
Lymphocyte count, × 10^9^/L	1.7 (1.5, 2.1)	1.5 (1.1, 1.9)	0.005
NLR	2.06 (1.7, 2.56)	2.63 (1.83, 3.505)	0.002
Hemoglobin, g/L	129.9 ± 17.7	127.5 ± 18.6	0.557
Albumin, g/L	40 (37, 42)	40.4 ± 4.7	0.383
Serum cystatin C (mg/L)	1.0 1 (0.875, 1.175)	1.195 (0.965, 1.385)	0.003
Serum uric acid (umol/L)	337.2 ± 97.1	364.5 ± 105.2	0.11
Total cholesterol, mmol/L	4.0 ± 1.0	4.1 ± 1.0	0.721
Triglyceride, mmol/L	1.35 (0.9875, 1.8825)	1.515 (1.1375, 2.395)	0.151
LDL, mmol/L	2.04 (1.7025, 2.805)	1.92 (1.6825, 2.575)	0.874
hs-TnI, pg/ml	17.15 (6.525, 82.625)	21.8 (4.7, 96.2)	0.917
BNP, pg/ml	118.2 (47.725, 482.175)	240.9 (108.3, 511.4)	0.041
Serum creatinine, umol/L	70.9 ± 16.8	78 (67, 98.5)	< 0.001
Anti-hypertensive drugs, *N* (%)			
ACEI	39 (40.2%)	21 (36.8%)	0.0679
ARB	11 (11.3%)	9 (15.8%)	0.428
Use of Aspirin, *N* (%)	94 (96.9%)	52 (91.2%)	0.247
Urine output during the cardiopulmonary bypass	900 (600, 1275)	1018 ± 563	0.803
Cardiopulmonary bypass time, min	114.5 (93, 125)	125 (108, 130)	0.004
Aortic clamping time, min	63 (53, 70.5)	68 (58.5, 81)	0.019
Preoperative LVEF (%)-no., %			< 0.001
≥ 50	66 (94.3%)	34 (70.8%)	
< 50	4 (5.7%)	14 (29.2%)	

*LVEF* left ventricular ejection fraction, *AKI* acute kidney injury, *NLR* neutrophil-to-lymphocyte ratio, *LDL* low-density lipoprotein, *CKD* chronic kidney disease, *MI* myocardial infarction, *AF* atrial fibrillation, *HF* heart failure, *hs-TnI* high-sensitivity troponin I

### Relationship between NLR and postoperative AKI after ONCAB

**Fig. 1:**
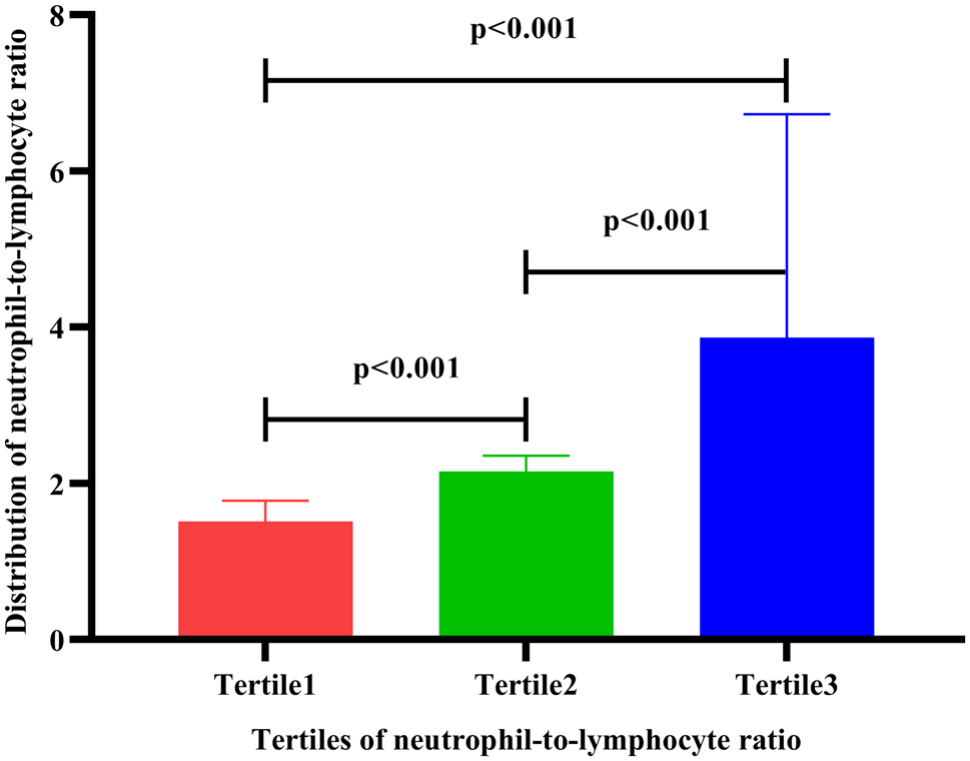
Neutrophil to lymphocyte ratio levels in on-pump coronary artery bypass patients who were grouped in tertiles

**Fig. 2:**
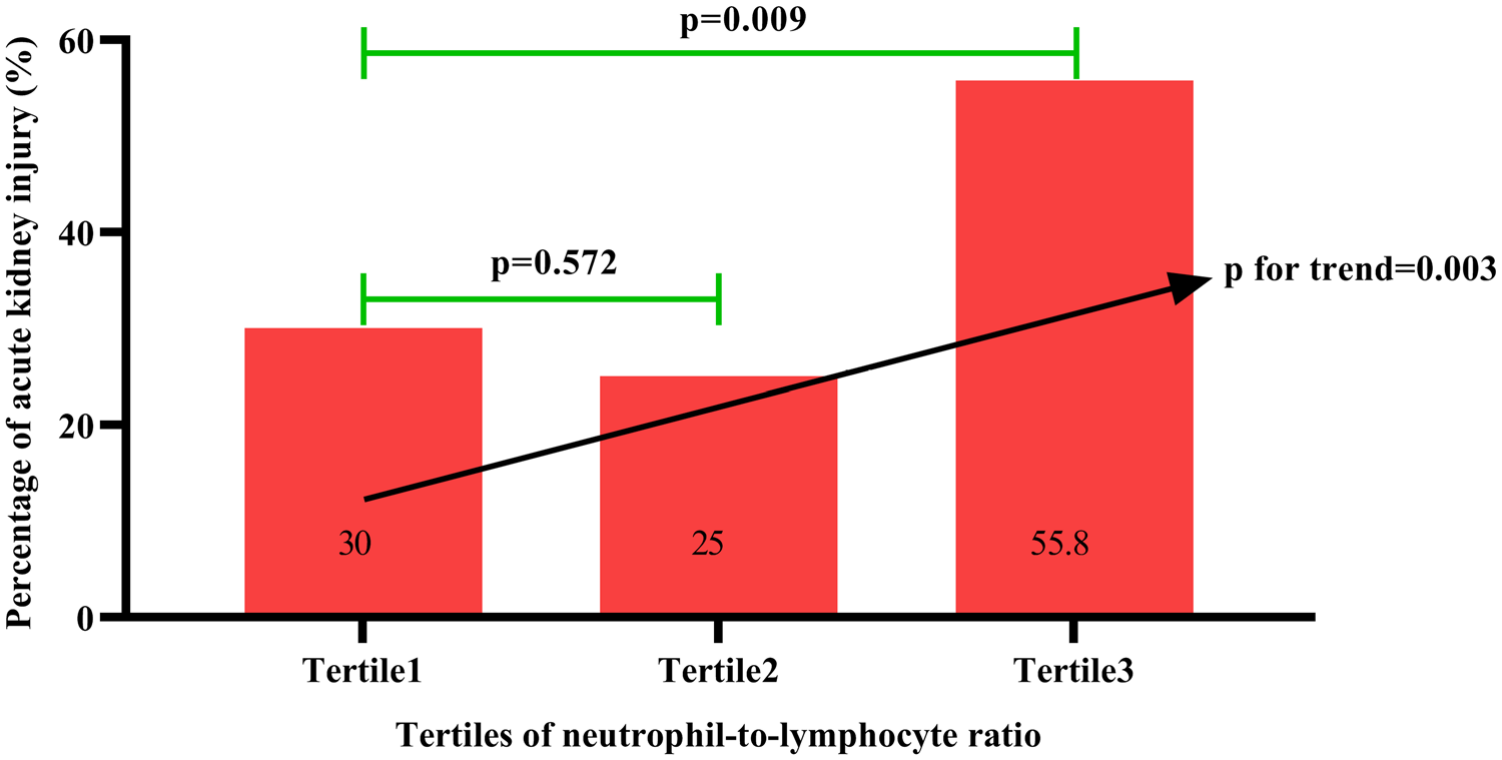
Neutrophil to lymphocyte ratio and rates of AKI. Tertile1, Tertile2 and Tertile3 are tertile groups based on NLR levels

To clarify the relationship between NLR and postoperative AKI after ONCAB, we further grouped the patients by the tertiles of NLR. The results showed that NLR was higher in patients in Tertile2 compared to those in Tertile1 (2.135 (2, 2.2475) vs. 1.585 (1.345, 1.7225), *p* < 0.001), also was significantly higher in Tertile3 compared to those in Tertile1 (3.04 (2.7925, 3.6925) vs. 1.585 (1.345, 1.7225), *p* < 0.001). Compared to patients in Tertile2, NLR was significantly higher in those in Tertile3 (2.135 (2, 2.2475) vs. 3.04 (2.7925, 3.6925), *p* < 0.001). The distribution of NLR in Tertiles is shown in Fig. 1. We also compared the clinical baseline data of the Tertiles. Leukocyte count, neutrophil count was higher in Tertile2 and Tertile3 patients compared with Tertile1 (*p* < 0.05), and lymphocyte count was higher in Tertile1 and Tertile2 patients compared to Tertile3 (*p* < 0.05). We also found a progressively higher percentage (30% vs. 25% vs. 55.8%) and risk of AKI after ONCAB in the three groups (*p* for trend = 0.003, Fig. 2). When we performed subgroup analysis for those who estimated glomerular filtration rate (eGFR) ≥ 60 ml/min/1.73 m^2^, this trend still exists, we found that after dividing patient into tertiles based on NLR, those with higher NLR had higher risk of postoperative AKI than those with lower NLR (28.3% vs. 24.4% vs. 50%, *p* for trend = 0.018).While there was no significant difference among the three groups in age, gender, or comorbidities, including diabetes, hypertension, atrial fibrillation, myocardial infarction, cerebrovascular disease, and heart failure (*p* > 0.05), and no significant differences (*p* > 0.05) were found among the three groups in hemoglobin, albumin, blood lipids, ultrasensitive troponin, serum Cystatin C, uric acid, BNP, cardiopulmonary bypass time, ACC time, and cardiac function, as shown in Table 2. Results in Table 1 showed that the occurrence of AKI in patients after ONCAB might be influenced by various aspects. Then we further included the history of chronic kidney disease, lymphocyte count, NLR, serum Cystatin C, serum creatinine, BNP, cardiopulmonary bypass time, ACC time, and LVEF in the multivariate logistic regression analysis, the results showed that patients with higher preoperative NLR had greater risk of AKI [odds ratio (OR) = 4.91, 95% confidential interval (CI) 1.45–16.58, *p* = 0.0104], and patients with higher preoperative serum creatinine also had greater risk of AKI after surgery (OR = 1.054, 95% CI 1.006–1.105, *p* = 0.026), as shown in Table 3.

**Table 2:** Characteristics of 154 patients with on-pump coronary artery bypass by preoperative NLR tertiles

Range of NLR	Tertile1	Tertile2	Tertile3	*P *value
	0.75–1.85	1.85–2.56	2.61–22.6	
	50	52	52	
Age-no., %				0.463
≦60 years	17 (34.0%)	21 (40.4%)	15 (28.8%)	
> 60 years	33 (66.0%)	31 (59.6%)	37 (71.2%)	
Gender-no., %				0.474
Male	31 (62.0%)	34 (65.4%)	38 (73.1%)	
Female	19 (38.0%)	18 (34.6%)	14 (26.9%)	
Diabetes-no., %				0.817
No	32 (64.0%)	31 (59.6%)	34 (65.4%)	
Yes	18 (36.0%)	21 (40.4%)	18 (34.6%)	
Hypertension-no., %				0.187
No	12 (24.0%)	18 (34.6%)	10 (19.2%)	
Yes	38 (76.0%)	34 (65.4%)	42 (80.8%)	
Previous AF-no., %				0.292
No	47 (94.0%)	46 (88.5%)	50 (96.2%)	
Yes	3 (6.0%)	6 (11.5%)	2 (3.8%)	
Previous Stroke-no., %				0.764
No	40 (80.0%)	43 (82.7%)	40 (76.9%)	
Yes	10 (20.0%)	9 (17.3%)	12 (23.1%)	
Previous MI-no., %				0.72
No	29 (58.0%)	26 (50.0%)	28 (53.8%)	
Yes	21 (42.0%)	26 (50.0%)	24 (46.2%)	
CKD-no., %				0.091
No	49 (98.0%)	50 (96.2%)	46 (88.5%)	
Yes	1 (2.0%)	2 (3.8%)	6 (11.5%)	
Previous HF-no., %				0.336
No	32 (64.0%)	40 (76.9%)	35 (67.3%)	
Yes	18 (36.0%)	12 (23.1%)	17 (32.7%)	
WBC count, × 10^9^/L	5.5 ± 1.4	6.2 ± 1.1	6.015 (5.3, 7.4)	0.003
Neutrophil count, × 10^9^/L	2.9 ± 0.9	3.8 ± 0.6	4.2 (3.7, 5.3)	< 0.001
Lymphocyte count, × 10^9^/L	2.0 ± 0.5	1.7 (1.6, 1.975)	1.2 (1.1, 1.5)	< 0.001
NLR	1.585 (1.345, 1.7225)	2.135 (2, 2.2475)	3.04 (2.7925, 3.6925)	< 0.001
Hemoglobin, g/L	127.2 ± 18.1	132.0 ± 15.2	127.8 ± 20.3	0.411
Albumin, g/L	40 (37, 43)	40.6 ± 4.1	40 (37, 42)	0.42
Serum cystatin C (mg/L)	1.08 (0.91, 1.2)	1.0 ± 0.3	1.2 ± 0.3	0.117
Serum uric acid (umol/L)	349.3 ± 93.2	315.5 (266.75, 382.75)	355.7 ± 91.9	0.314
Total cholesterol, mmol/L	4.1 ± 1.2	4.1 ± 0.8	3.4 (3.0525, 4.2475)	0.135
Triglyceride, mmol/L	1.33 (1.01, 1.85)	1.51 (1.01, 2.39)	1.405 (0.995, 1.965)	0.693
LDL, mmol/L	1.95 (1.6, 2.885)	2.31 (1.92, 2.77)	1.84 (1.5275, 2.51)	0.059
hs-TnI, pg/ml	27.65 (8.175, 76.475)	11.5 (5.775, 54.475)	23.9 (4.3, 123.05)	0.285
BNP, pg/ml	126 (61.7, 477.2)	113.95 (44.775, 408.8)	225.15 (99.55, 583.15)	0.161
Serum creatinine, umol/L	75.5 (68, 90.25)	69.8 ± 17.4	73 (63.25, 90.5)	0.027
Anti-hypertensive drugs, *N* (%)				
ACEI	20 (40%)	16 (30.8%)	24 (46.2%)	0.27
ARB	8 (16%)	5 (9.6%)	7 (13.5%)	0.627
Use of aspirin, *N* (%)	49 (98%)	50 (96.2%)	47 (90.4%)	0.20
Urine output during the cardiopulmonary bypass	1000 (600, 1300)	1000 (600, 1500)	900 (500, 1200)	0.512
Cardiopulmonary bypass time, min	124 (103.75, 130.25)	113.5 (90, 125.75)	121 (100, 126)	0.069
Aortic clamping time, min	65 (58, 80)	65.5 (54, 70.75)	63.8 ± 16.8	0.427
AKI-no., %				0.002
NO	35 (70.0%)	39 (75.0%)	23 (44.2%)	
YES	15 (30.0%)	13 (25.0%)	29 (55.8%)	
Preoperative LVEF (%)-no., %				0.092
≥ 50	35 (92.1%)	35 (87.5%)	30 (75.0%)	
< 50	3 (7.9%)	5 (12.5%)	10 (25.0%)	

*LVEF* left ventricular ejection fraction, *AKI* acute kidney injury, *NLR* neutrophil-to-lymphocyte ratio, *LDL* low-density lipoprotein, *CKD* chronic kidney disease, *MI* myocardial infarction, *AF* atrial fibrillation, *HF* heart failure, *hs-TnI* high-sensitivity troponin I, *CABG* coronary artery bypass graft

**Table 3:** Binary logistic regression of AKI

Variable	Univariable		Multivariable	
	OR (95% CI)	*P*	OR (95% CI)	*P*
CKD(no = 0, yes = 1)	6.6 (1.3–33.2)	0.021	1.12 (0.052–24.44)	0.940
Lymphocyte count, × 10^9^/L	0.3 (0.2–0.7)	0.005	0.79 (0.12–5.15)	0.802
NLR	2.0 (1.3–2.9)	< 0.001	4.91 (1.45–16.58)	0.0104
Serum cystatin C (mg/L)	6.9 (1.7–27.8)	0.006	0.48 (0.01–22.82)	0.708
BNP, pg/ml	1.0 (1.0–1.0)	0.161	0.999 (0.997–1.001)	0.406
Serum creatinine, umol/L	1.0 (1.0–1.1)	< 0.001	1.054 (1.006–1.105)	0.026
Cardiopulmonary bypass time, min	1.0 (1.0–1.0)	0.004	1.034 (0.976–1.095)	0.255
Aortic clamping time, min	1.0 (1.0–1.0)	0.021	1.001 (0.943–1.063)	0.965
LVEF (%) (0 ≥ 50%, 1 < 50%)	6.8 (2.1–22.2)	0.002	3.751 (0.682–20.638)	0.129

*LVEF* left ventricular ejection fraction, *AKI* acute kidney injury, *NLR* neutrophil-to-lymphocyte ratio, *CKD* chronic kidney disease

### Predictive value of NLR for the occurrence of AKI after ONCAB

**Fig. 3:**
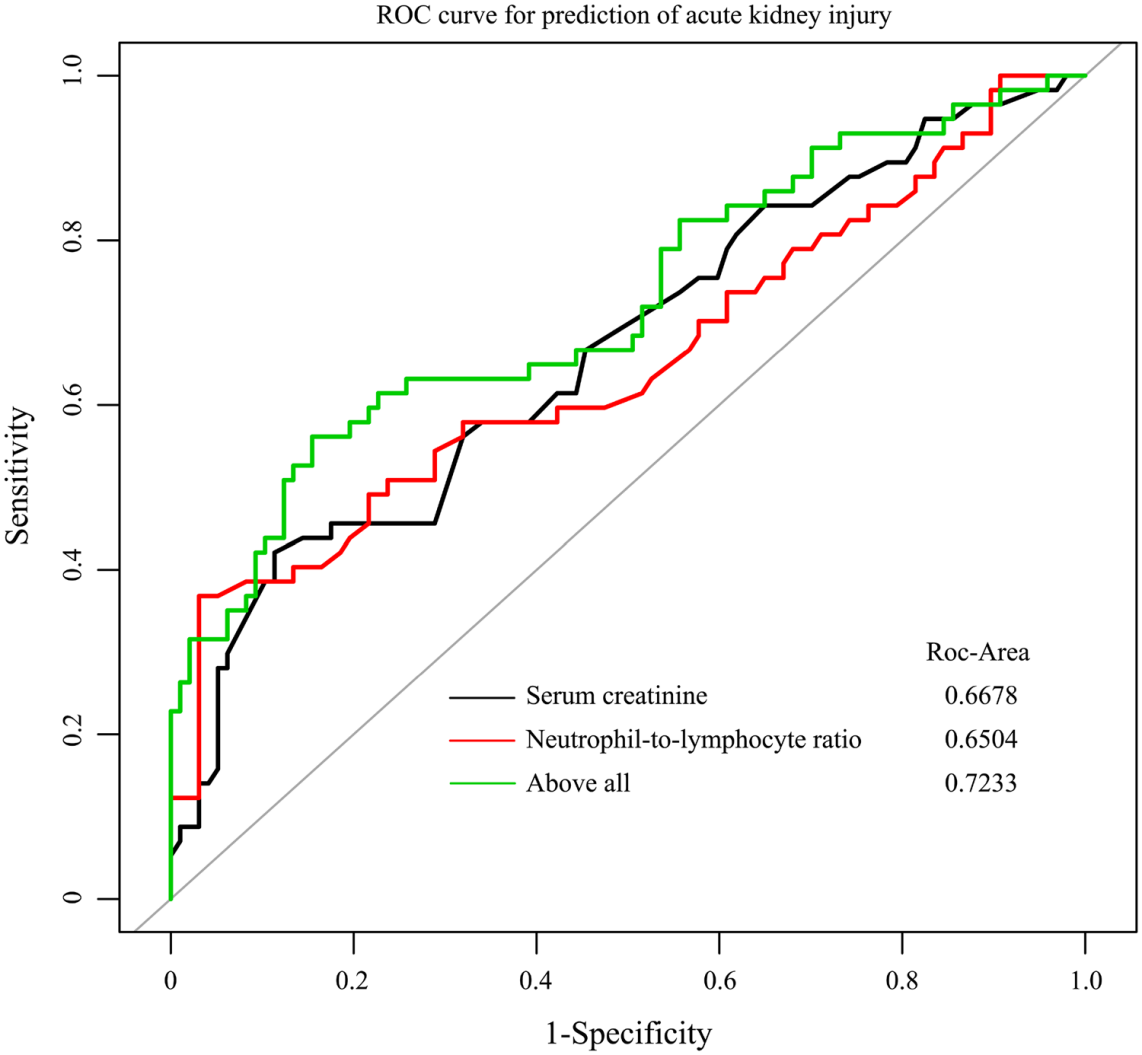
Receiver operating characteristic curve (ROC) for the prediction of AKI

To clarify the predictive value of NLR for the occurrence of AKI after ONCAB, we performed the analysis using the receiver operator characteristic (ROC) curve, which showed that the area under the curve (AUC) of NLR to predict AKI after ONCAB was 0.6504 (95% CI 0.555–0.7457) with the high specificity of 96.91% but the sensitivity of nearly 36.84%, and the cut-off value was 3.09, this similar trend also exists for those who eGFR more than 60 ml/min/1.73 m^2^, as shown in Tables 3 and 4. Both univariate and multivariate analyses showed that patients’ preoperative serum creatinine levels (AUC was 0.6678, 95% CI 0.5772–0.7583, cut-off value was 87. 5 umol/L) was also significantly associated with the incidence of postoperative AKI, so the diagnostic efficacy was significantly improved when preoperative NLR and serum creatinine were introduced simultaneously for the combined diagnosis, and the AUC for the diagnosis of postoperative incidence of AKI increased to 0.7233 (95% CI 0.6364–0.8101), with the specificity of 84.54% and the sensitivity of 56.14%, as shown in Fig. 3.

**Table 4:** Binary logistic regression analysis of AKI with variables and the AUC and optimal threshold of each related variable for those who eGFR ≥ 60 ml/min/1.73 m^2^

Variable	Univariable regression analysis		AUC and optimal threshold	
	OR (95% CI)	*P*	AUC(95% CI)	Optimal threshold
CKD(no = 0, yes = 1)	1.0 (0.1–10.8)	0.971	–	–
Lymphocyte count, × 10^9^/L	0.3 (0.1–0.8)	0.009	0.63 (0.53–0.74)	1.25
NLR	1.9 (1.3–2.9)	0.002	0.64(0.54–0.75)	3.09
Serum cystatin C (mg/L)	5.9 (1.0–33.8)	0.048	0.63 (0.51–0.75)	1.13
Cardiopulmonary bypass time, min	1.0 (1.0–1.0)	0.006	0.65 (0.55–0.75)	114.5
Aortic clamping time, min	1.0 (1.0–1.0)	0.044	0.60 (0.50–0.70)	69.5

*eGFR* estimated glomerular filtration rate, *AKI* acute kidney injury, *NLR* neutrophil-to-lymphocyte ratio, *CKD* chronic kidney disease, *AUC* the area under the curve

## Discussion

This study retrospectively analyzed the association between preoperative NLR and the incidence of AKI after ONCAB in 154 cases. The results showed that preoperative NLR > 3.09 was an independent predictor of postoperative AKI. We also found that patients with high preoperative serum creatinine levels (> 87.5 umol/L) indicated higher risk of postoperative AKI.

CABG is one of the treatments for coronary atherosclerotic heart disease. In the United States in 2018, 181, 000 cases underwent CABG accounting for 63% of all cardiac surgeries, while in China in 2019 nearly 50, 000 cases CABG [11]. AKI associated with cardiac surgery is a common and serious complication of cardiac surgery under extracorporeal circulation, with some studies showing even up to 40.5% [12]. This also suggests that many patients undergoing CABG may have postoperative AKI without early warning and interventional treatment. Compared to healthy human, AKI patients have 8.8-fold increased risk of progression to chronic kidney disease and 3.3-fold increased risk of progression to end-stage renal disease (ESRD), in addition, more than one-third of patients with AKI in long-term follow-up progress to chronic kidney disease or ESRD [13]. Currently, serum creatinine and urine volume remain the main indicators for the diagnosis of AKI, however, these two indicators often have the lag feature and are of limited value in early diagnosis [14]. Therefore, it is necessary to find ideal biomarkers for the diagnosis of AKI. Unlike neutrophil gelatinase-associated lipocalin (NGAL), interleukin-18, insulin-like growth factor binding protein 7 antibody (IGFBP7), etc., NLR is the combination of neutrophil and lymphocyte counts that is economical and easily available and can be performed in various hospitals. Since inflammation plays an important role in the pathophysiology of AKI, NLR as an inflammatory marker may have the ability to predict AKI [15].

Previous studies have found that alterations in neutrophil and lymphocyte counts and postoperative NLR imbalance are strongly associated with severity of coronary artery disease, poor long-term prognosis, and postoperative complications [16, 17]. Similar to previous research studies, our study showed that increased preoperative NLR was associated with AKI after ONCAB [18]. Increasing neutrophil count is an indicator of active inflammatory process, whereas decreasing lymphocyte count is an indicator of inadequate active inflammatory process. However, individual leukocyte parameter is susceptible to alteration by external conditions (e.g., dehydration, hemodilution, etc.), and NLR is relatively more stable. Elevated NLR reflects the chronic inflammatory state of the body that is exacerbated by extracorporeal circulation and surgery. Although NLR can be obtained simply by routine blood test, there is no consensus to date on the threshold value for NLR. Previous studies suggested the preoperative NLR threshold of 2.65 for predicting AKI after ONCAB alone [18]; similarly, in the present study we found the significant increase in the incidence of AKI with the preoperative NLR > 3.09 after adjusting for confounding factors, which still needs confirmation by large sample studies in the future.

The present study also found that preoperative serum creatinine levels directly influenced the incidence of postoperative AKI. Our results showed that preoperative serum creatinine was significantly higher in patients in the AKI group compared with those in non-AKI group and was positively correlated with the highest blood creatinine value, suggesting that the earlier the onset and the greater the elevation of blood creatinine, the greater the likelihood of AKI. After adjusting other risk factors, high preoperative serum creatinine levels also significantly increased the risk of postoperative AKI, which was consistent with previous studies [8, 19, 20]. Previous studies showed that preoperative serum creatinine ≥ 1.3 mg/dl was an independent risk factor for AKI associated with cardiac surgery, which was largely similar to our results [21].

As one of the important indicators of renal function, increasing serum creatinine before surgery to some extent suggests the concomitant renal insufficiency or reduced renal compensatory capacity, and is prone to induce postoperative hemodynamic flocculation, inflammatory factor release and ischemia–reperfusion injury due to the complexity of CABG, which can further deteriorate renal function. Therefore, it will be of great positive significance for improving prognosis to active monitor renal function after CABG, timely detect abnormal serum creatinine, take appropriate renal protection measures, stop nephrotoxic drugs as early as possible, and correct factors affecting renal perfusion.

Previous studies showed that prolonged cardiopulmonary bypass time and ACC time were also independent risk factors for postoperative complications of AKI in patients undergoing ONCAB [22, 23]. Since renal blood flow and perfusion are relatively insufficient during extracorporeal circulation, massive erythrocytes were destroyed, and the released free hemoglobin consequently caused tubular obstruction and reduced glomerular rate filtration. In addition, blood can also be diluted in extracorporeal circulation and lead to reduced oxygen uptake and renal damage [24–26]. Similar to other studies [27], after adjusting confounding factors we did not find a correlation between the cardiopulmonary bypass time and postoperative AKI. This may be related to the short cardiopulmonary bypass time in these patients compared to other studies, which still needs to be confirmed by more studies at a later stage.

There are many limitations in the present research: (i) this is a single-center retrospective study with a small sample size, there is a publication bias, so the results might not be directly extrapolated to other patient groups; (ii) data on C-reactive protein, a common inflammatory index were not collected; (iii) the history of chronic kidney disease were based on self review of patients, and may underestimate the actual incidence rate; (iv) the characteristics of vascular lesions, combined surgery and other factors that may affect the prognosis of surgery were not analyzed. Larger trials are warranted to further substantiate the findings of the present study.

In conclusion, our study showed a high incidence of AKI after ONCAB. An elevated preoperative NLR (> 3.09) and serum creatinine (> 87.5 umol/L) are independent risk factors for postoperative AKI and can serve as an early warning. Early intervention is needed in high-risk patients to reduce the occurrence of AKI to improve long-term prognosis.

## Supplementary Information

Below is the link to the electronic supplementary material.

Supplementary file1 (DOCX 39 KB)

## Data Availability

All data generated or analyzed during this study are included in this article. Further enquiries can be directed to the corresponding author.
