# Supplementary material for: Predictive value of neutrophil to lymphocyte ratio on acute kidney injury after on-pump coronary artery bypass: a retrospective, single-center study
Source: Gen Thorac Cardiovasc Surg. 2022 Feb 1;70(7):624–33. doi: 10.1007/s11748-022-01772-z (PMC9206599; doi:10.1007/s11748-022-01772-z)
Supplement: Supplementary file 1 — Supplementary file1 (DOCX 39 KB) [file 11748_2022_1772_MOESM1_ESM.docx]

| **Table1 Demographic and Clinical Characteristics of the Patients at Baseline for those who eGFR≥60ml/min/1.73m^2^** | | | |
| --- | --- | --- | --- |
| Group | No-AKI | AKI | P-value |
|  | 90 | 47 |  |
| Age-no., % |  |  |  |
| ≦60 years | 31（34.4%） | 19（40.4%） | 0.49 |
| >60 years | 59（65.6%） | 28（59.6%） |  |
| Gender-no., % |  |  | 0.233 |
| Male | 58（64.4%） | 35（74.5%） |  |
| Female | 32（35.6%） | 12（25.5%） |  |
| Diabetes-no., % |  |  | 0.15 |
| NO | 56（62.2%） | 35（74.5%） |  |
| YES | 34（37.8%） | 12（25.5%） |  |
| Hypertension-no., % |  |  | 0.413 |
| NO | 27（30%） | 11（23.4%） |  |
| YES | 63（70%） | 36（76.6%） |  |
| Previous AF-no., % |  |  | 0.763 |
| NO | 83（92.2%） | 44（93.6%） |  |
| Yes | 7（7.8%） | 3（6.4%） |  |
| Previous Stroke-no., % |  |  | 0.673 |
| NO | 72（80%） | 39（83%） |  |
| YES | 18（20%） | 8（17%） |  |
| Previous MI-no., % |  |  | 0.979 |
| NO | 50（55.6%） | 26（55.3%） |  |
| YES | 40（44.4%） | 21（44.7%） |  |
| CKD-no., % |  |  | 0.971 |
| NO | 88（97.8%） | 46（97.9%） |  |
| YES | 2（2.2%） | 1（2.1%） |  |
| Previous HF-no., % |  |  | 0.447 |
| NO | 65（72.2%） | 31（66%） |  |
| YES | 25（27.8%） | 16（34%） |  |
| WBC count, ×10^9^/L | 5.96 ± 1.38 | 5.88（5.3,6.78） | 0.754 |
| Neutrophil count, ×10^9^/L | 3.66 ± 1.04 | 3.9（3.1,4.6） | 0.198 |
| Lymphocyte count, ×10^9^/L | 1.7（1.4,2.02） | 1.5 ± 0.5 | 0.01 |
| NLR | 2.09（1.73,2.64） | 2.63（1.88,3.5） | 0.006 |
| Hemoglobin, g/L | 129 ± 18 | 130 ± 18 | 0.972 |
| Albumin, g/L | 40（37,42） | 41 ± 4.6 | 0.078 |
| Serum Cystatin C (mg/L) | 1（0.87,1.15） | 1.12 ± 0.26 | 0.031 |
| Serum uric acid (umol/L) | 329 ± 91 | 351 ± 97 | 0.878 |
| Total cholesterol, mmol/L | 3.92 ± 0.97 | 4.19 ± 1.05 | 0.869 |
| Triglyceride, mmol/L | 1.25（0.98,1.85） | 1.58（1.14,2.5） | 0.071 |
| LDL, mmol/L | 2.01（1.73,2.82） | 1.98（1.74,2.79） | 0.722 |
| hs-TnI, pg/ml | 14.2（6.3,66.3） | 18.3（4.45,98.75） | 0.796 |
| BNP, pg/ml | 113.05（47.1，448.73） | 209.4（85.15，445.4） | 0.107 |
| Serum creatinine, umol/L | 68 ± 13 | 76 ± 16 | 0.11 |
| Anti-hypertensive drugs, *N* (%) |  |  |  |
| ACEI | 35（38.9%） | 19（40.4%） | 0.861 |
| ARB | 10（11.1%） | 5（10.6%） | 0.933 |
| Use of Aspirin, *N* (%) | 87（96.7%） | 42（89.4%） | 0.178 |
| Urine output during the cardiopulmonary bypass | 900（600，1225） | 1064 ± 550 | 0.468 |
| Cardiopulmonary bypass time,min | 113（93,126） | 125（108,130） | 0.004 |
| Aortic clamping time, min | 72 ± 25 | 63（53,69） | 0.05 |
| Preoperative LVEF (%)-no., % |  |  | 0.014 |
| ≥50 | 61（93.8%） | 30（75%） |  |
| <50 | 4（6.2%） | 10（25%） |  |
| LVEF:left ventricular ejection fraction, AKI：acute kidney injury, NLR:neutrophil-to-lymphocyte ratio, LDL:low-density lipoprotein, CKD:chronic kidney disease, MI:myocardial infarction, AF:atrial fibrillation, HF:heart failure, hs-TnI:high-sensitivity troponin I | | | |

| **Table2 Characteristics of 137 patients with on-pump coronary artery bypass by preoperative NLR tertiles for those who eGFR≥60ml/min/1.73m^2^** | | | | |
| --- | --- | --- | --- | --- |
| Range of NLR | Tertile1 | Tertile2 | Tertile3 | P-value |
|  | 0.89-1.89 | 1.91-2.63 | 2.64-22.6 |  |
|  | 46 | 45 | 46 |  |
| Age-no., % |  |  |  | 0.365 |
| ≦60 years | 16（34.8%） | 20（44.4%） | 14（30.4%） |  |
| >60 years | 30（65.2%） | 25（55.6%） | 32（69.6%） |  |
| Gender-no., % |  |  |  | 0.661 |
| Male | 29（63%） | 31（68.9%） | 33（71.7%） |  |
| Female | 17（37%） | 14（31.1%） | 13（28.3%） |  |
| Diabetes-no., % |  |  |  | 0.616 |
| NO | 30（65.2%） | 28（62.2%） | 33（71.7%） |  |
| YES | 16（34.8%） | 17（37.8%） | 13（28.3%） |  |
| Hypertension-no., % |  |  |  | 0.536 |
| NO | 14（30.4%） | 14（31.1%） | 10（21.7%） |  |
| YES | 32（69.6%） | 31（68.9%） | 36（78.3%） |  |
| Previous AF-no., % |  |  |  | 0.478 |
| NO | 41（89.1%） | 42（93.3%） | 44（95.7%） |  |
| Yes | 5（10.9%） | 3（6.7%） | 2（4.3%） |  |
| Previous Stroke-no., % |  |  |  | 0.969 |
| NO | 37（80.4%） | 37（82.2%） | 37（80.4%） |  |
| YES | 9（19.6%） | 8（17.8%） | 9（19.6%） |  |
| Previous MI-no., % |  |  |  | 0.94 |
| NO | 26（56.5%） | 24（53.3%） | 26（56.5%） |  |
| YES | 20（43.5%） | 21（46.7%） | 20（43.5%） |  |
| CKD-no., % |  |  |  | 0.156 |
| NO | 46（100%） | 44（97.8%） | 44（95.7%） |  |
| YES | 0 | 1（2.2%） | 2（4.3%） |  |
| Previous HF-no., % |  |  |  | 0.137 |
| NO | 28（60.9%） | 36（80%） | 32（69.6%） |  |
| YES | 18（39.1%） | 9（20%） | 14（30.4%） |  |
| WBC count, ×10^9^/L | 5.5 ± 1.5 | 6.2 ± 1.1 | 6.0（5.2,7.1） | 0.013 |
| Neutrophil count, ×10^9^/L | 2.8（2.3,3.7） | 3.9 ± 0.7 | 4.2（3.6,5.3） | <0.001 |
| Lymphocyte count, ×10^9^/L | 1.9 ± 0.5 | 1.7（1.6,1.9） | 1.2（1.1,1.5） | <0.001 |
| NLR | 1.6（1.4,1.8） | 2.2（2.1,2.4） | 3.0（2.8,3.7） | <0.001 |
| Hemoglobin, g/L | 127 ± 18 | 133 ± 15 | 134（115,143） | 0.376 |
| Albumin, g/L | 39.5（37,43.3） | 40.8 ± 4.2 | 40（37,42） | 0.559 |
| Serum Cystatin C (mg/L) | 1.08 ± 0.23 | 0.99 ± 0.21 | 1.09 ± 0.25 | 0.128 |
| Serum uric acid (umol/L) | 330.9 ± 78.3 | 334.6 ± 111.3 | 343.6 ± 88.5 | 0.799 |
| Total cholesterol, mmol/L | 4.1 ± 1.1 | 4.1 ± 0.9 | 3.4（2.9,4.2） | 0.157 |
| Triglyceride, mmol/L | 1.3（1.0,1.7） | 1.6（1.0,2.4） | 1.4（0.9,2.0） | 0.332 |
| LDL, mmol/L | 2.4 ± 1.1 | 2.0（1.8,2.8） | 1.9（1.5,2.6） | 0.291 |
| hs-TnI, pg/ml | 21.8（4.8,44.6） | 11.8（6.2,70） | 23.9（3.8,133.3） | 0.546 |
| BNP, pg/ml | 122.4（49,432） | 106.6（48.6,405.1） | 225.2（82.3,596.9） | 0.128 |
| Serum creatinine, umol/L | 72.5 ± 12.9 | 68.0 ± 16.0 | 72.0 ± 14.9 | 0.272 |
| Anti-hypertensive drugs, *N* (%) |  |  |  |  |
| ACEI | 16（34.8%） | 16（35.6%） | 22（47.8%） | 0.358 |
| ARB | 5（10.9%） | 5（11.1%） | 5（10.9%） | 0.999 |
| Use of Aspirin, *N* (%) | 44（95.7%） | 44（97.8%） | 41（89.1%） | 0.185 |
| Urine output during the cardiopulmonary bypass | 1000（600,1300） | 1000（725,1500） | 874 ± 455 | 0.148 |
| Cardiopulmonary bypass time,min | 124（100,130） | 118（90,126） | 122（101,126） | 0.184 |
| Aortic clamping time, min | 62.5（54.8,80） | 65（53.5,70） | 64.5 ± 16.9 | 0.944 |
| AKI-no., % |  |  |  | 0.021 |
| NO | 33（71.7%） | 34（75.6%） | 23（50%） |  |
| YES | 13（28.3%） | 11（24.4%） | 23（50%） |  |
| Preoperative LVEF (%)-no., % |  |  |  | 0.353 |
| ≥50 | 32（91.4%） | 31（88.6%） | 28（80%） |  |
| <50 | 3（8.6%） | 4（11.4） | 7（20%） |  |
| LVEF:left ventricular ejection fraction, AKI：acute kidney injury, NLR:neutrophil-to-lymphocyte ratio, LDL:low-density lipoprotein, CKD:chronic kidney disease, MI:myocardial infarction, AF:atrial fibrillation, HF:heart failure, hs-TnI:high-sensitivity troponin I, CABG: coronary artery bypass graft | | | | |

| **Table3 Binary Logistic regression analysis of AKI with variables and the AUC and optimal threshold of each related variable for those who eGFR≥60ml/min/1.73m^2^** | | | | |
| --- | --- | --- | --- | --- |
| Variable | Univariable regression analysis | | AUC and optimal threshold | |
|  | OR(95%CI) | P | AUC(95%CI) | Optimal threshold |
| CKD(no=0,yes=1) | 1.0 (0.1-10.8) | 0.971 | - | - |
| Lymphocyte count, ×10^9^/L | 0.3 (0.1-0.8) | 0.009 | 0.63 (0.53-0.74) | 1.25 |
| NLR | 1.9 (1.3-2.9) | 0.002 | 0.64(0.54-0.75) | 3.09 |
| Serum Cystatin C (mg/L) | 5.9 (1.0-33.8) | 0.048 | 0.63 (0.51-0.75) | 1.13 |
| Cardiopulmonary bypass time,min | 1.0 (1.0-1.0) | 0.006 | 0.65 (0.55-0.75) | 114.5 |
| Aortic clamping time, min | 1.0 (1.0-1.0) | 0.044 | 0.60 (0.50-0.70) | 69.5 |
| eGFR: Estimated Glomerular Filtration Rate, AKI：acute kidney injury, NLR:neutrophil-to- lympho  cyte ratio, CKD:chronic kidney disease,AUC:the area under the curve. | | | | |
